# A review of risk assessment for driving in rainy weather

**Published:** 2024-10

**Authors:** Akram Ahmadizadeh Fini, Elham Ahmadizadeh Fini

**Affiliations:** ^*a*^ Student Research Committee, Shiraz University of Medical Sciences, Shiraz, Iran.; ^*b*^ Social Determinants in Health Promotion Research Center, Hormozgan University of Medical Sciences, Bandar Abbas, Iran.; ^*c*^ Student Research Committee, Hormozgan University of Medical Sciences, Bandar Abbas, Iran.

## Abstract

**Background:**

Driving in rainy weather is considered one of the most challenging conditions due to potential hazards. Four key factors—human, vehicle, road, and environment—are always involved in accidents, with environmental factors and climatic phenomena playing a significant role. Therefore, this study aims to review and evaluate several studies conducted in this area as well as the main risk factors associated with driving in rainy conditions.

**Methods:**

This article focuses on the risk factors associated with driving in adverse weather conditions, which include varying intensities of rainfall. Through a comprehensive search of electronic databases using the keywords "risk analysis," "driving," "rainfall," and their equivalents, relevant sources were identified. After reviewing the studies found, key and practical insights were presented.

**Results:**

These findings highlight the complexity of human behavior in response to adverse weather conditions, particularly during rainfall, where the perceived risk and past experiences play a crucial role in decision-making. Survey results from similar incidents indicate that the following groups of individuals have the highest percentage of driving accidents during rainfall: 1. Individuals aged 18 to 35. 2. Those who do not have accurate information about flooded roads 3. People who have not previously experienced flooding 4. Demographic factors also play a significant role.

**Conclusions:**

This study elucidates the complex and interrelated impact of weather characteristics, pavement conditions, road safety, and various demographic factors on driving accidents in rainy weather. Unlike other incidents that show a decline in mortality statistics, unfortunately, there is no substantial evidence reported regarding fatalities related to rainfall and the resulting floods. Although there is limited direct study assessing the main risk factors associated with driving in floodwaters, numerous reports highlight the varied reactions of individuals in such conditions. Each report emphasizes, to varying degrees, the importance of the interaction between environmental information, social processes, and individual factors.

**Keywords:**

Risk analysis, Driving, Rainy weather

